# Identification of a Two-m6A RNA Methylation Regulator Risk Signature as an Independent Prognostic Biomarker in Papillary Renal Cell Carcinoma by Bioinformatic Analysis

**DOI:** 10.1155/2021/4582082

**Published:** 2021-02-06

**Authors:** Feilong Yang, Guojiang Zhao, Liyuan Ge, Yimeng Song, Kai Hong, Hongxian Zhang, Cheng Liu, Peng Hong, Lulin Ma

**Affiliations:** Department of Urology, Peking University Third Hospital, Beijing, China

## Abstract

N6-Methyladenosine (m6A), the most common form of mRNA modification, is dynamically regulated by the m6A RNA methylation regulators, which play an important role in regulating the gene expression and phenotype in both health and disease. However, the role of m6A in papillary renal cell carcinoma (pRCC) is unknown. The purpose of this work is to investigate the prognostic value of m6A RNA methylation regulators in pRCC; thus, we can build a risk score model based on m6A RNA methylation regulators as a risk signature for predicting the prognosis of pRCC. Here, we investigated the expression and corresponding clinical data by bioinformatic analysis based on 289 pRCC tissues and 32 normal kidney tissues obtained from TCGA database. As a result, we identified the landscape of m6A RNA methylation regulators in pRCC. We grouped all pRCC patients into two clusters by consensus clustering to m6A RNA methylation regulators, but we found that the clusters were not correlated to the prognosis and clinicopathological features of pRCC. Therefore, we additionally built a two-m6A RNA methylation regulator risk score model as a risk signature by the univariate Cox regression analysis and least absolute shrinkage and selection operator (LASSO) Cox regression. The risk signature was constructed as follows: 0.031HNRNPC + 0.199KIAA1429. It revealed that the risk score was associated with the clinicopathological features such as pT status and pN status of pRCC. More importantly, the risk score was an independent prognostic marker for pRCC patients. Thus, m6A RNA methylation regulators contributed to the malignant progression of pRCC influencing its prognosis.

## 1. Introduction

The papillary renal cell carcinoma (pRCC) subtype accounts for approximately 10-20% of RCC which is a kind of lethal cancers in the genitourinary system [1–3]. Currently, radical or partial nephrectomies are the main and most effective therapeutic options for clinical localized and locally advanced pRCC. However, a number of patients develop recurrent or metastatic diseases that significantly reduce the overall survival (OS) of patients. Although clinicopathological features can provide clues to evaluate the prognosis of pRCC, it is barely enough as the biological heterogeneity is a hallmark of human cancers [4]. Generally, tumor genotype variations among tumors within different patients are known as interpatient heterogeneity, and variability among multiple tumors of the same type arising in the same patient is referred to as intrapatient heterogeneity, and the subpopulation of cancer cells with distinct phenotypic and molecular features within a tumor is regarded as intratumor heterogeneity [4, 5]. The clinicopathological features do not reflect these biological heterogeneities. Therefore, novel independent prognostic biomarkers are helpful to predict the prognosis of pRCC.

RNA methylation modification accounts for more than 60% of the 171 known RNA posttranscriptional modifications which play a fundamental role in many biological and disease processes [6–8]. N6-Methyladenosine (m6A) modification is the methylation of the adenosine base at the nitrogen-6 position, which is the most prevalent internal mRNA modification in mammals [9–11]. The m6A methylation site appears in the nuclear RNA under the action of methyltransferases, also known as “writers,” mainly including KIAA1429, METTL3, METTL14, RBM15, WTAP, and ZC3H13, which are responsible for the addition of the methyl group to the nitrogen on the sixth carbon of the aromatic ring of an adenosine residue [8, 12, 13]. The m6A RNA methylation site in the nucleus can also be erased under the action of demethylases, known as “erasers,” mainly including ALKBH5 and FTO that can specifically target m6A RNA [8, 13–15]. Subsequently, in the further processing of RNA in the nucleus and outside the nucleus, the m6A binding proteins, also known as “readers,” mainly including HNRNPC, YTHDC1, YTHDC2, YTHDF1, and YTHDF2, will bind to the m6A methylated site to perform a series of biological functions in regulating the stability, localization, transportation, translation, and degradation of target mRNAs that depended on the recognition of different kinds of “readers” [6, 8, 9, 13, 16–19]. Therefore, the methylation level of m6A and the expression level of m6A RNA methylation regulators may have important roles in the pathogenesis and clinical management of pRCC.

Growing evidence has proved that m6A modification plays a critical role in gene expression regulation, as well as the carcinogenesis, progression, and prognosis of various human malignancies [6, 20]. However, little is known about the function of m6A-related genes in pRCC. In the present study, we systematically investigated the role of the 13 widely reported m6A-related proteins in pRCC based on the RNA-seq transcriptome and corresponding clinical datasets of the pRCC cohort from The Cancer Genome Atlas (TCGA) database by bioinformatic analysis. As a result, we found the expression landscape of m6A RNA methylation regulators in pRCC and built a two-m6A RNA methylation regulator risk score model as a risk signature to independently predict the prognosis of pRCC.

## 2. Materials and Methods

### 2.1. The mRNA Expression and Clinical Datasets of pRCC

The RNA-seq transcriptome and corresponding clinical datasets of the pRCC cohort were downloaded from TCGA Research Network (https://cancergenome.nih.gov/) [21]. The version of the dataset was Data Release 24.0-May 07, 2020. All mRNASeq gene expression matrix and corresponding clinical data of the samples were extracted via Perl (version: strawberry-perl-5.30.2.1-64bit for Windows). The extracted matrix is used for subsequent bioinformatic analysis. A total of 32 cancer-adjacent normal tissues and 289 cancer tissues from patients with pRCC were included. Perl, R (version: 4.0.1 for Windows), RStudio (version: 1.1.456 for Windows), and corresponding R packages were used for subsequent bioinformatic analysis.

### 2.2. Differential Expression Analysis and Correlation Analysis of m6A RNA Methylation Regulators

The expression matrix of the 13 m6A RNA methylation regulators was extracted from all mRNASeq gene expression matrix data. To investigate the function of m6A RNA methylation regulators in pRCC, the “limma” [22] package was utilized to screen differentially expressed m6A RNA methylation regulators in pRCC tissues compared with normal kidney tissues. *p* < 0.05 was considered statistically significant. The heatmap and vioplot were performed, respectively, by the “pheatmap” package (http://cran.r-project.org/web/packages/pheatmap/index.html) and the “vioplot” package (https://cran.r-project.org/web/packages/vioplot/index.html) visualizing the expression levels and differences of the 13 m6A RNA methylation regulators in pRCC. Furthermore, to identify the potential correlation of the 13 m6A RNA methylation regulators, Spearman correlation analysis was performed by the “corrplot” package (https://cran.r-project.org/web/packages/corrplot/index.html).

### 2.3. Identification of Consensus Clusters of m6A RNA Methylation Regulators

To identify whether the cancer tissues can be grouped into different clusters based on the expression similarity of m6A RNA methylation regulators, we grouped 289 cancer tissues using the “ConsensusClusterPlus” package (http://www.bioconductor.org/packages/release/bioc/html/ConsensusClusterPlus.html). Moreover, to verify the results of the grouping, PCA was performed using the “ggplot2” package (http://cran.r-project.org/web/packages/ggplot2/index.html).

### 2.4. OS and Clinicopathological Characteristics of pRCC in Clusters

To identify the difference in the OS rate in the clusters, the K-M survival analysis of the clusters was performed using the “survival” package (http://cran.r-project.org/web/packages/survival/index.html). Besides, we also investigated the expression of the 13 m6A RNA methylation regulators and the clinicopathological features of pRCC in clusters using the “pheatmap” package.

### 2.5. Building the Risk Signature with m6A RNA Methylation Regulators in pRCC

To better understand the prognostic value of m6A RNA methylation regulators in pRCC, firstly, a univariate Cox regression analysis was performed based on the expression levels of m6A RNA methylation regulators and corresponding clinical survival data using the “survival” package. Then, the statistically significant m6A RNA methylation regulators were used for the least absolute shrinkage and selection operator (LASSO) Cox regression [23, 24]. The LASSO algorithm was performed by the “glmnet” package (https://cran.r-project.org/web/packages/glmnet/index.html) and the “survival” package. Based on the minimum criteria and the coefficients obtained from the LASSO algorithm, it would select appropriate m6A RNA methylation regulators to build the risk signature to calculate the risk score for pRCC patients [25]. Based on the median cutoff point of the risk score, a risk score model was constructed, in which all the patients were divided into the high-risk group and low-risk group. Then, K-M survival analysis was performed for the two groups using the “survival” package to identify whether the prognostic model was statistically significant. A *p* < 0.05 was considered to indicate a statistically significant difference.

### 2.6. Identification of the Relationship between the Risk Score and the Clinicopathological Characteristics

To better understand the role of the risk score in the clinical outcomes of pRCC, we systematically investigated the relationship between the risk score and the clinicopathological characteristics by the “pheatmap” package, including age, gender, stage status, pT status, pM status, and pN status. We also investigated the relationship between the risk score and the expression levels of the selected risk signatures using the “pheatmap” package. Furthermore, to verify the predictive accuracy of the risk score, a receiver operating characteristic (ROC) curve of 5-year survival rates for pRCC patients was performed and the area under the curve (AUC) was calculated using the “survival” package and “timeROC” package (http://cran.r-project.org/web/packages/timeROC/index.html). Besides, to determine whether the risk score was an independent prognostic indicator for pRCC patients, we performed univariate and multivariate Cox regression analyses using the “survival” package and “forestplot” package (https://cran.r-project.org/web/packages/forestplot/index.html). The confounding factors included age, gender, stage status, pT status, pM status, and pN status. A *p* < 0.05 was statistically significant.

## 3. Results

### 3.1. The Landscape of m6A RNA Methylation Regulators in pRCC

**Figure 1:**
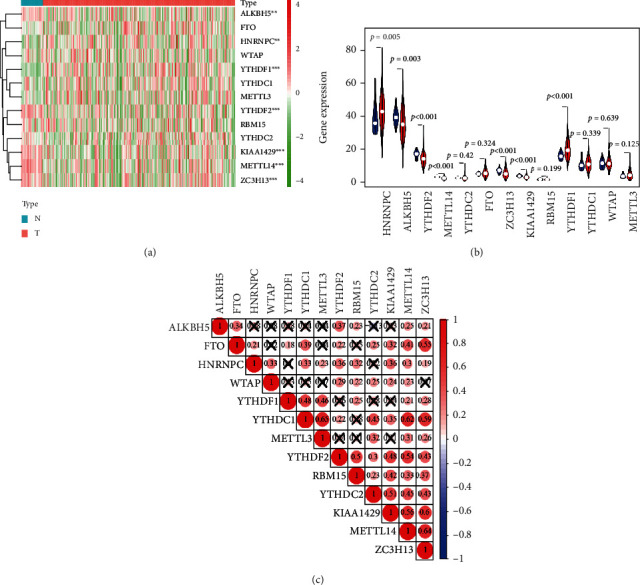
The landscape of m6A RNA methylation regulators in pRCC. (a) Expression levels of 13 m6A RNA methylation regulators in pRCC. The red is upregulated, and the green is downregulated. The higher or lower the expression, the darker the color. The upper tree diagram represents clustering results for different samples from different experimental groups, and the left tree shows cluster analysis results for different genes from different samples. (b) The vioplot visualizing the differentially expressed m6A RNA methylation regulators in pRCC. The blue represents normal kidney tissues, and the red represents pRCC tissues. (c) Spearman correlation analysis of the 13 m6A RNA methylation regulators in pRCC. pRCC: papillary renal cell carcinoma; ^∗^*p* < 0.05, ^∗∗^*p* < 0.01, and ^∗∗∗^*p* < 0.001.

There were 13 genes included in the landscape of m6A RNA methylation regulators in pRCC. Firstly, we compared the expression levels of the 13 m6A RNA methylation regulators in 289 pRCC tissues and 32 normal kidney tissues obtained from TCGA dataset. It revealed that the expression levels of HNRNPC and YTHDF1 were higher, while the expression levels of ALKBH5, YTHDF2, KIAA1429, METTL14, and ZC3H13 were lower in pRCC tissues compared with normal kidney tissues (Figures 1(a) and 1(b)). In addition, as shown in Figure 1(c), the relationship between the 13 m6A RNA methylation regulators was positively correlated. The correlation was weak to moderate, and METTL14 and ZC3H13 were the most relevant genes (Figure 1(c)).

### 3.2. Two Clusters of pRCC Identified by Consensus Clustering of m6A RNA Methylation Regulators

**Figure 2:**
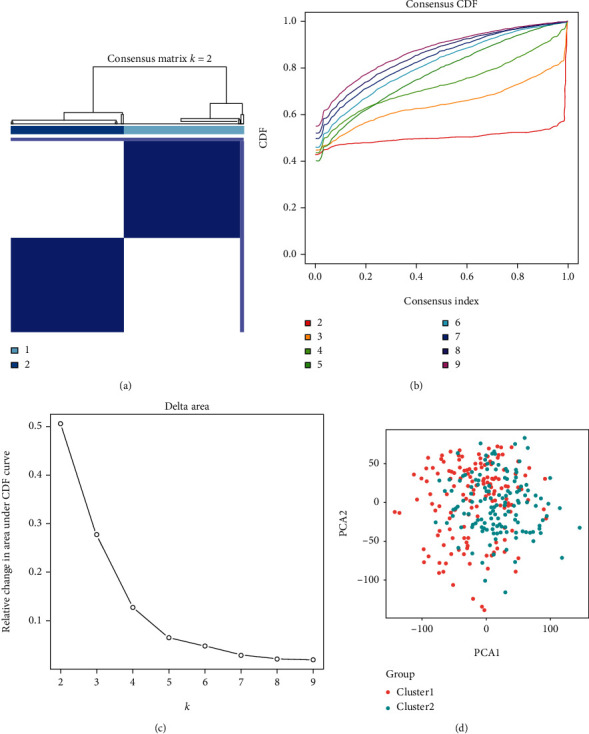
Identification of consensus clusters by m6A RNA methylation regulators in pRCC. (a) Consensus clustering matrix for *k* = 2. (b) Consensus clustering CDF for *k* = 2-9. (c) Relative change in area under the CDF curve for *k* = 2-9. (d) PCA of the 289 pRCC tissues from TCGA dataset. pRCC patients in cluster 1 and cluster 2 did not gather together. pRCC: papillary renal cell carcinoma; CDF: cumulative distribution function; PCA: principal component analysis.

Based on the expression similarity of m6A RNA methylation regulators, we grouped the 289 pRCC tissues into two clusters (Figure 2(a)). For *k* = 2, it seemed that the CDF value was smaller (Figures 2(b) and 2(c)). Then, PCA was performed to identify whether our classification was correct, and the results showed that cluster 1 and cluster 2 did not gather together clearly (Figure 2(d)). These findings indicated that the clusters of pRCC by m6A RNA methylation regulators might be suspicious, and it needed more investigations.

### 3.3. The Clusters of pRCC Were Not Correlated to OS Rates and Clinicopathological Features

**Figure 3:**
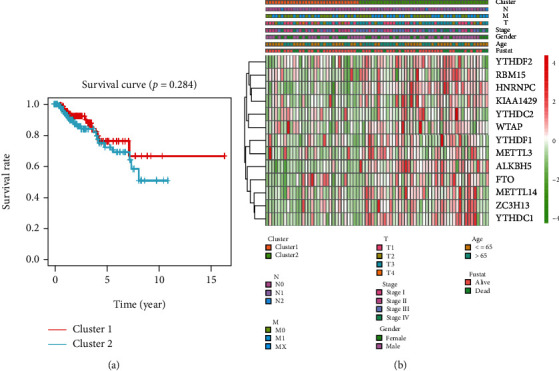
Differential clinicopathological features and OS of pRCC in the two clusters. (a) Kaplan-Meier OS curves for the two clusters (cluster 1/2) of pRCC patients. It revealed that clusters of pRCC were not correlated to the OS rates. (b) Heatmap and clinicopathological features of the two clusters (cluster 1/2). The red is upregulated, and the green is downregulated. The higher or lower the expression, the darker the color. It revealed that clusters of pRCC were not correlated to the clinicopathological features. The upper tree diagram represents clustering results for different samples from different experimental groups, and the left tree shows cluster analysis results for different genes from different samples. pRCC: papillary renal cell carcinoma; OS: overall survival.

Although our classification by consensus clustering of m6A RNA methylation regulators might not be correct, we still investigated the relationship between the clusters and the OS rates, as well as the clinicopathological features, including age, gender, stage status, pT status, pM status, and pN status. As a result, it revealed that clusters of pRCC were not correlated to the OS rates and clinicopathological features (Figures 3(a) and 3(b)).

### 3.4. A Two-m6A-Related Gene Risk Score Model Was Identified in pRCC

**Figure 4:**
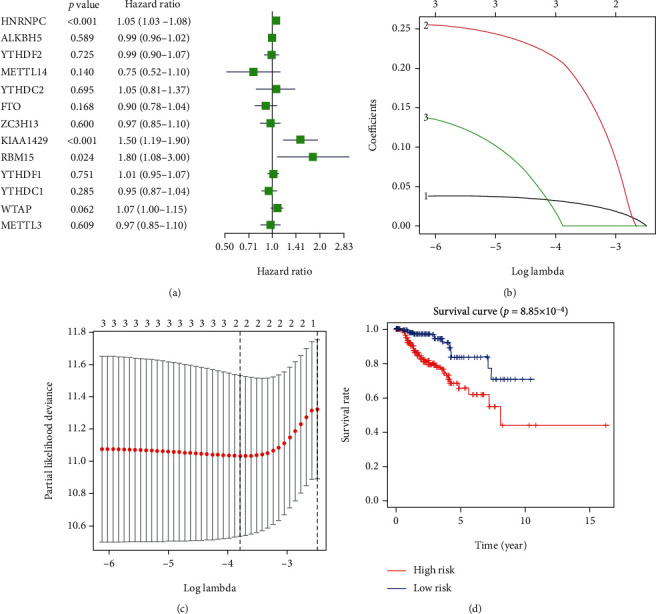
Risk signature of pRCC with two m6A RNA methylation regulators. (a) The screening process of m6A RNA methylation regulators involved in later LASSO regression by univariate Cox regression. There were 3 m6A RNA methylation regulators screened out. (b, c) The coefficients and variable selection using the LASSO model. Two m6A RNA methylation regulators were selected to build the risk score model as a risk signature. (d) Kaplan-Meier OS curves for patients in the high- and low-risk groups based on the risk score. pRCC: papillary renal cell carcinoma; LASSO: least absolute shrinkage and selection operator; OS: overall survival.

To better investigate the prognostic value of m6A RNA methylation regulators in pRCC, we performed a univariate Cox regression analysis based on the expression levels of m6A RNA methylation regulators and corresponding clinical survival data. It indicated that high expression of HNRNPC (HR = 1.05, 95% CI = 1.03-1.08), KIAA1429 (HR = 1.50, 95% CI = 1.19-1.90), and RBM15 (HR = 0.80, 95% CI = 1.08-3.00) had a worse OS rate in patients with pRCC (Figure 4(a)). The LASSO Cox regression algorithm was applied to build a risk score model considering 3 m6A RNA methylation regulators (HNRNPC, KIAA1429, and RBM15) which were associated with worse clinical outcomes by univariate Cox regression analysis. Then, two genes (HNRNPC and KIAA1429) were selected to build the risk signature to calculate the risk score for pRCC patients (Figures 4(b) and 4(c)). The risk signature was constructed as follows: 0.031HNRNPC + 0.199KIAA1429. Based on the median cutoff point (7.772) of the risk score, a two-m6A-related gene risk score model was constructed, in which all the patients were divided into the high-risk group and low-risk group. The number of events (death) was 40 of 283 patients, and the median (min-max) duration with OS was 1.85 (0.01-16.23) years. The result indicated that the high-risk group had a worse OS rate in patients with pRCC (Figure 4(d)).

### 3.5. Risk Score Was Associated with Clinicopathological Features and Could Serve as an Independent Prognostic Marker in pRCC

**Figure 5:**
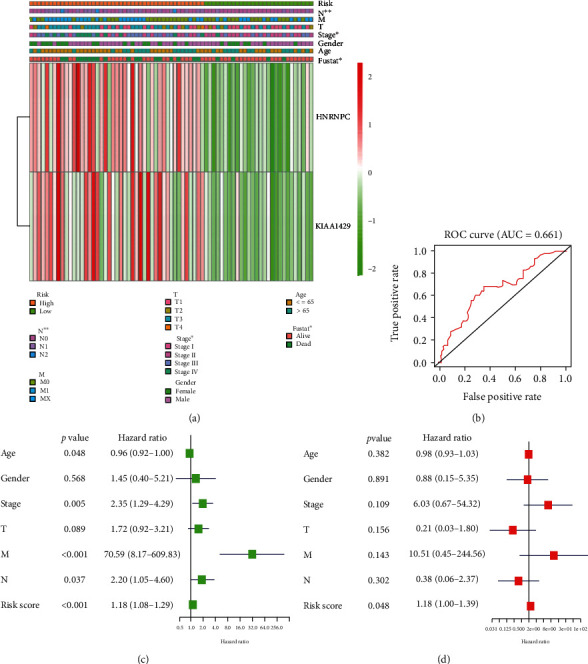
Relationship between the risk score and the clinicopathological features, as well as OS of pRCC. (a) Relationship between the risk score and the clinicopathological features. The heatmap showed the expression levels of the two m6A RNA methylation regulators in low- and high-risk pRCC patients. It also indicated that the risk score was closely correlated to pT status and pN status of pRCC patients. (b) ROC curves showed the predictive efficiency of the risk signature. (c) Univariate Cox regression analyses of the clinicopathological features (including the risk score). It showed that the risk score, age, stage status, pM status, and pN status were closely correlated to the prognosis of pRCC patients. (d) Multivariate Cox regression analyses of the clinicopathological features (including the risk score). It indicated that the risk score was an independent prognostic biomarker for pRCC patients. pRCC: papillary renal cell carcinoma; ROC: receiver operating characteristic; OS: overall survival; ^∗^*p* < 0.05 and ^∗∗^*p* < 0.01.

Investigating the relationship between the risk score and the clinicopathological characteristics, it was revealed that the risk score was closely correlated to pT status and pN status of pRCC patients, and the high risk was generally along with the higher pT status and pN status (Figure 5(a)). Moreover, compared with the low-risk group patients, pRCC patients generally contained a higher proportion of HNRNPC and KIAA1429 in the high-risk group (Figure 5(a)). In addition, the result of the ROC curve indicated that the risk score could predict 5-year OS rates for pRCC patients (AUC = 0.661) (Figure 5(b)). Next, investigating whether the risk score was an independent prognostic indicator, it was shown that the risk score, age, stage status, pM status, and pN status were closely correlated to the prognosis of pRCC (Figure 5(c)). However, it indicated that only a risk score could be an independent prognostic marker for pRCC patients (HR = 1.18, 95% CI = 1.00-1.39) (Figure 5(d)).

## 4. Discussion

The m6A RNA methylation is dynamically regulated by 13 widely reported m6A RNA methylation regulators, which are widely distributed in various types of RNA, such as mRNA, transport RNA (tRNA), and ribosomal RNA (rRNA), and play an important role in regulating splicing, stability, localization, transportation, and translation of target mRNAs in the posttranscriptional level [6, 26, 27]. As the most abundant internal modification in eukaryotic mRNAs, m6A RNA methylation plays a critical role not only in various normal biological processes but also in the initiation, progression, and even drug responses of different types of cancers [6, 9, 10, 28–30]. As reported recently, m6A RNA methylation is not “good or bad” in the study of m6A and tumors, because it can promote or suppress cancer cells mainly by regulating the mRNA expression of related oncogenes or tumor suppressor genes [25]. The dysregulation of m6A regulators can affect cell proliferation, cell self-renewal and differentiation capacity, control of heat shock response [31], DNA damage response [32], tissue development [33], cell death, and development of multiple forms of human diseases, including cancer [8, 11, 19, 34, 35]. Emerging evidence has demonstrated that m6A RNA methylation regulators are also involved in the occurrence and development of many cancers: hepatocellular carcinoma [36, 37], glioblastoma [35], osteosarcoma [38], colorectal cancer [39], and so on [19]. However, the role of m6A RNA methylation regulators in pRCC remains unknown.

This study attempted to investigate the role of the 13 widely reported m6A RNA methylation regulators in the prognosis of pRCC. Firstly, we described the expression landscape of the 13 m6A RNA methylation regulators in 289 pRCC tissues compared with 32 normal kidney tissues. There were 2 highly expressed regulators (HNRNPC and YTHDF1) and 5 lowly expressed genes (ALKBH5, YTHDF2, KIAA1429, METTL14, and ZC3H13) in pRCC tissues. The correlation between the 13 m6A RNA methylation regulators was positively correlated, and the correlation was weak to moderate, and METTL14 and ZC3H13 were the most relevant. More importantly, we constructed a risk signature (risk score = 0.031HNRNPC + 0.199KIAA1429) by using 2 m6A RNA methylation regulators, including HNRNPC and KIAA1429. Based on the median cutoff point (7.772) of the risk score, we divided all patients into the high-risk group and low-risk group. The result of the univariate Cox regression analysis indicated that the risk score, age, stage status, pM status, and pN status were closely correlated to the prognostics of pRCC. And the result of the multivariate Cox regression analysis showed that the risk score was an independent prognostic factor for pRCC patients (HR = 1.18, 95% CI = 1.00-1.39).

Up to now, the role and specific mechanism of m6A RNA methylation regulators are not fully understood and lack specificity. Though we have demonstrated the prognostic values of m6A RNA methylation regulators in pRCC patients, their accurate mechanism in the progression and prognosis of pRCC remains unclear. Recently, using TCGA database, several similar studies provided us with clues to understand the role and mechanism of the m6A RNA methylation regulators. In gastric cancer, a risk signature was constructed using 3 m6A RNA methylation regulators (FTO, RBM15, and ALKBH5), which not only was an independent prognostic marker but could also predict the clinicopathological features of gastric cancer [25]. In bladder cancer, it was found that m6A RNA methylation regulators could participate in the malignant progression of bladder cancer, and a risk signature with three selected m6A RNA methylation regulators (FTO, YTHDC1, and WTAP) could serve as a promising prognostic biomarker [8]. In ccRCC, it was also reported that a three-gene risk signature, including METTL3, METTL14, and HNRNPA2B1, or a risk signature with two m6A methylation regulators (METTL3 and METTL14) not only was an independent prognostic factor but was also closely associated with clinicopathological features [40–42]. In our study, we constructed the risk signature with HNRNPC and KIAA1429. KIAA1429 was an interacting partner of the methyltransferase complex components [43]. The expression level of KIAA1429 was significantly higher in breast cancer and hepatocellular carcinoma, and high expression of KIAA1429 could promote cell proliferation and metastasis in breast cancer in vivo and in vitro [37, 44]. The high expression of KIAA1429 was also significantly related to poor OS of hepatocellular carcinoma [37]. HNRNPC was highly expressed in bladder cancer, and the HNRNPC knockdown reduced the proliferation of breast cancer cells [8, 45]. In our study, we found that KIAA1429 was lowly expressed, while HNRNPC was highly expressed in pRCC tissues, and these two m6A-related genes were of great significance in the prognosis of pRCC. Therefore, modulating m6A RNA methylation modifications might be a novel strategy to guide the targeted therapy for pRCC patients. Unfortunately, there were several limitations in our study. Firstly, the expression levels of the m6A RNA methylation regulators were not verified in our pRCC samples. Secondly, although we investigated the role of the m6A RNA methylation regulators in the prognosis of pRCC, especially HNRNPC and KIAA1429, we did not further explore their accurate mechanism. In our future study, we will verify the result using our own clinical pRCC samples utilizing m6A sequencing, real-time quantitative PCR, immunohistochemistry, and so on. In addition, we will investigate the mechanism of m6A RNA methylation regulators influencing the progression and prognosis of pRCC, especially HNRNPC and KIAA1429. It deserves more investigations.

## 5. Conclusion

In summary, our results systematically demonstrated the expression and prognostic value of m6A RNA methylation regulators in pRCC. Using HNRNPC and KIAA1429, we constructed a two-m6A methylation regulator risk score model as a risk signature, which could serve as an independent prognostic biomarker to predict the prognosis of pRCC patients.

## Data Availability

The RNA-seq transcriptome data and corresponding clinicopathological information of the pRCC cohort in this study were obtained from the public database TCGA (https://cancergenome.nih.gov/). The version of the dataset was Data Release 24.0-May 07, 2020. The data format of Gene Expression Quantification was HTSeq-FPKM. The data format of the corresponding clinical data was BCR XML.
